# Abnormalities of Cortical Sources of Resting State Delta Electroencephalographic Rhythms Are Related to Epileptiform Activity in Patients With Amnesic Mild Cognitive Impairment Not Due to Alzheimer's Disease

**DOI:** 10.3389/fneur.2020.514136

**Published:** 2020-10-23

**Authors:** Claudio Babiloni, Giuseppe Noce, Carlo Di Bonaventura, Roberta Lizio, Maria Teresa Pascarelli, Federico Tucci, Andrea Soricelli, Raffaele Ferri, Flavio Nobili, Francesco Famà, Eleonora Palma, Pierangelo Cifelli, Moira Marizzoni, Fabrizio Stocchi, Giovanni B. Frisoni, Claudio Del Percio

**Affiliations:** ^1^Department of Physiology and Pharmacology “Vittorio Erspamer”, Sapienza University of Rome, Rome, Italy; ^2^San Raffaele Cassino, Cassino (FR), Italy; ^3^IRCCS SDN, Naples, Italy; ^4^Epilepsy Unit, Department of Neurosciences/Mental Health, Sapienza University of Rome, Rome, Italy; ^5^Oasi Research Institute - IRCCS, Troina, Italy; ^6^Department of Motor Sciences and Healthiness, University of Naples Parthenope, Naples, Italy; ^7^Clinica Neurologica, IRCCS Ospedale Policlinico San Martino, Genova, Italy; ^8^Dipartimento di Neuroscienze, Oftalmologia, Genetica, Riabilitazione e Scienze Materno-infantili (DiNOGMI), Università di Genova, Genova, Italy; ^9^Pasteur Institute-Cenci Bolognetti Foundation, Rome, Italy; ^10^IRCCS Neuromed, Pozzilli, Italy; ^11^Scienze Cliniche Applicate e Biotecnologiche, University of L'Aquila, L'Aquila, Italy; ^12^Laboratory of Alzheimer's Neuroimaging and Epidemiology, IRCCS Istituto Centro San Giovanni di Dio Fatebenefratelli, Brescia, Italy; ^13^IRCCS San Raffaele Pisana, Rome, Italy; ^14^Memory Clinic and LANVIE-Laboratory of Neuroimaging of Aging, University Hospitals and University of Geneva, Geneva, Switzerland

**Keywords:** amnesic mild cognitive impairment (aMCI), dementia, alzheheimer's disease, exact low-resolution brain electromagnetic source tomography (eLORETA), resting state electroencephalographic (rsEEG) rhythms, epileptiform electroencephalographic activity (EEA)

## Abstract

In the present exploratory and retrospective study, we hypothesized that cortical sources of resting state eyes-closed electroencephalographic (rsEEG) rhythms might be more abnormal in patients with epileptiform EEG activity (spike-sharp wave discharges, giant spikes) and amnesic mild cognitive impairment not due to Alzheimer's disease (noADMCI-EEA) than matched noADMCI patients without EEA (noADMCI-noEEA). Clinical, neuroimaging, neuropsychological, and rsEEG data in 32 noADMCI and 30 normal elderly (Nold) subjects were available in a national archive. Age, gender, and education were carefully matched among them. No subject had received a clinical diagnosis of epilepsy. Individual alpha frequency peak (IAF) was used to determine the delta, theta, and alpha frequency bands of rsEEG rhythms. Fixed beta and gamma bands were also considered. Regional rsEEG cortical sources were estimated by eLORETA freeware. Area under receiver operating characteristic (AUROC) curves indexed the accuracy of eLORETA solutions in the classification between noADMCI-EEA and noADMCI-noEEA individuals. As novel findings, EEA was observed in 41% of noADMCI patients. Furthermore, these noADMCI-EEA patients showed higher temporal delta source activities as compared to noADMCI-no EEA patients and Nold subjects. Those activities discriminated individuals of the two NoADMCI groups with an accuracy of about 70%. The significant percentage of noADMCI-EEA patients showing EEA and marked abnormalities in temporal rsEEG rhythms at delta frequencies suggest a substantial role of underlying neural hypersynchronization mechanisms in their brain dysfunctions.

## Introduction

In most patients with sporadic late-onset Alzheimer's disease with dementia (ADD), episodic memory disorders are prominent. They are associated with both brain amyloidosis and neurodegenerative neuropathology mainly affecting basal forebrain, hippocampus, and posterior cerebral cortex (1). ADD patients also exhibit a much higher incidence of convulsive epileptic seizures than the age-matched general population do (2, 3). Furthermore, electroencephalographic (EEG) recordings have unveiled that ADD patients may present signs of subclinical, non-convulsive, and epileptiform EEG activity (EEA) including spike-sharp wave discharges, giant spikes, etc. (4–6). Concerning the EEA prevalence, 62% of ADD patients showed it in EEG recordings lasting 24 h (5) and only 3% of them in few minutes of eyes-closed rsEEG recordings using 19 scalp electrodes (4). Furthermore, about 50% of ADD patients exhibited EEA by high-resolution resting state magnetoencephalographic recordings lasting 1 h [>100 sensors; (7)].

More recently, in unpublished data of our research group we detected EEA in 25% of ADMCI patients by EEG recordings lasting 30 min even if none of them had previously received a clinical diagnosis of epilepsy or previous report of seizures or epileptiform EEG patterns. In relation to the ADMCI patients without EEA, those with EEA also showed more abnormalities in cortical sources of resting state eyes-closed electroencephalographic (rsEEG) rhythms at central, parietal, occipital, and temporal delta band (<4 Hz) as well as temporal theta band (4–8 Hz). These findings may reflect a pronounced derangement of neurophysiological low-frequency oscillatory mechanisms underpinning cortical arousal and quiet vigilance in ADMCI-EEA patients.

In the present exploratory and retrospective study on archive data, we used the same methodology to investigate whether compared with patients with amnesic MCI not due to AD and without epileptiform EEG activity (noADMCI-noEEA), those with EEA (noADMCI-EEA) may be characterized by greater abnormalities in the cortical rsEEG rhythms at the delta and theta bands.

## Materials and Methods

### Subjects and Diagnostic Criteria

**Figure 1:**
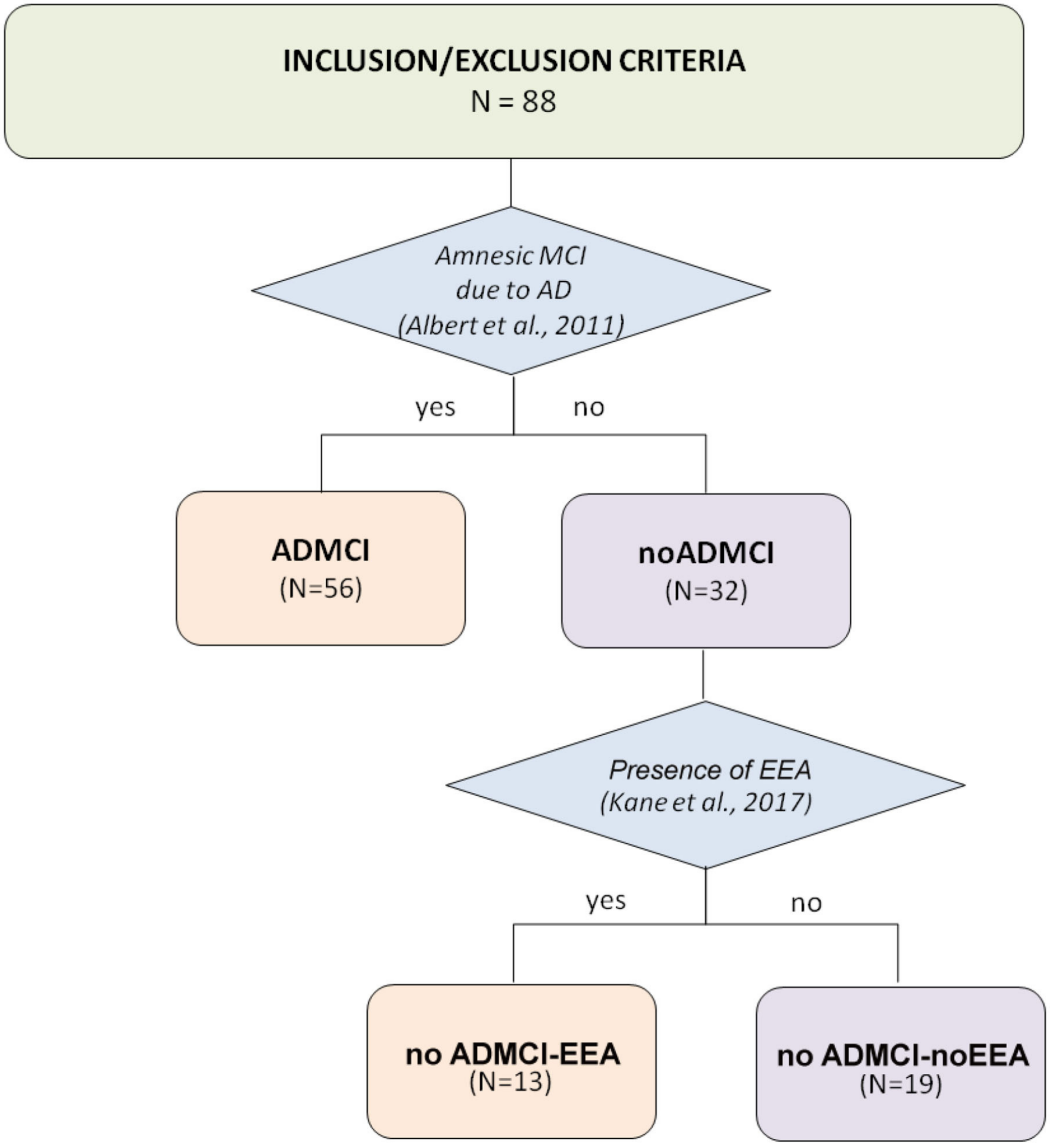
Flow chart illustrating the selection of patients with mild cognitive impairment not due to Alzheimer's disease (noADMCI) from the national (Italian) database of the present Consortium. This archive included 88 amnesic MCI patients: 56 patients with mild cognitive impairment due to Alzheimer's disease (ADMCI), and 32 noADMCI patients. These amnesic MCI patients were recruited by the following qualified Italian clinical units: the Sapienza University of Rome, Institute for Research and Evidence-based Cure (IRCCS) Fatebenefratelli of Brescia, IRCCS SDN of Naples, IRCCS Oasi Maria SS of Troina, University of Genova, Hospital San Raffaele of Cassino, and IRCCS San Raffaele Pisana of Rome.

Here we used the data of a national archive, formed by clinical, neuropsychological, anthropometric, genetic, cerebrospinal fluid (CSF), magnetic resonance imaging (MRI), and EEG data in 30 normal elderly subjects (Nold; 6 males; percentage of male: 20%) and 32 ADMCI patients (6 males; percentage of male: 18.8%). These subjects were recruited by the following Italian clinical units: Sapienza University of Rome, Institute for Research and Evidence-based Care (IRCCS) “Fatebenefratelli” of Brescia, IRCCS SDN of Naples, IRCCS Oasi Maria SS of Troina, University of Genova, Hospital San Raffaele of Cassino, and IRCCS San Raffaele Pisana of Rome. Figure 1 illustrates the flowchart of noADMCI patient selection from the mentioned national database, including ADMCI and noADMCI datasets.

Local institutional Ethics Committee approved the present observational study. All experiments were performed with the informed and overt consent of each participant or caregiver, in line with the Code of Ethics of the World Medical Association (Declaration of Helsinki) and the standards established by the local Institutional Review Board.

The inclusion criteria of the noADMCI patients were as follows: (1) age of 55–90 years; (2) reported memory complaints confirmed by a relative; (3) MMSE score ≥24; (4) Clinical Dementia Rating score of 0.5 [CDR; (8)]; (5) logical memory test (9) score of 1.5 standard deviation (SD) lower than the mean adjusted as age; (6) cognitive deficits not so strong to interfere significantly with the functional independence in the activities of the daily living; (7) Geriatric Depression Scale [15-item GDS; (10)] score of ≤5; (8) modified Hachinski ischemia (11) score of ≤4; (9) education of ≥5 years; (10) single amnesic or multi-domain (including amnesic) MCI status; and (11) amyloid beta 1-42 (i.e., Aβ 42) level in the cerebrospinal fluid (CSF) higher than 550 pg/mL (12).

The exclusion criteria of the noADMCI patients were as follows: (1) the “positivity” to one or more of the biomarkers of AD such as Aβ1-42/phospho-tau in the cerebrospinal fluid (CSF), FDG-positron emission tomography mapping, and structural magnetic resonance imaging (MRI) of the hippocampus, parietal, temporal, and posterior cingulate regions (13). This “positivity” was based on the judgment of “abnormality” of the readout given by physicians in charge for the diagnosis of patients, according to the local diagnostic routine of the participating clinical Units; (2) diagnosis of ADD according to the criteria of the Diagnostic and Statistical Manual of Mental Disorders, fourth edition (DSM-IV-TR; American Psychiatric Association) and the National Institute of Neurological Disorders and Stroke–Alzheimer Disease and Related Disorders (NINCDS–ADRDA) working group (14); (3) actual participation to a clinical trial using AD-modifying drugs; (4) mixed dementia including AD; (5) diagnosis of major psychiatric disorders (i.e., depression, etc.) or neurological illness not related to cognitive deficits; (6) diagnosis of epilepsy or report of seizures or epileptiform EEG activity in the past; (7) use of antiepileptics; or (8) chronic use of neuroleptics, narcotics, analgesics, sedatives, or hypnotics.

In all noADMCI patients, Apolipoprotein E (i.e., APOE) genotyping, and CSF biomarkers were assessed. CSF was pre-processed, frozen, and stored in line with the Alzheimer's Association Quality Control Programme for CSF biomarkers (15). Levels of amyloid beta 1-42 (i.e., Aβ42), protein tau (i.e., total tau, t-tau), and phosphorylated form of tau (i.e., p-tau) were also measured. Furthermore, anthropometric features (i.e., weight, height, and body mass index) and cardiocirculatory markers (i.e., systolic pressure, diastolic pressure, pulse pressure, mean arterial pressure, and heart frequency) were also taken in consideration in the analysis.

In all noADMCI patients, the following neuropsychological tests were assessed: (1) the global cognitive status was tested by the mini mental state evaluation exam (MMSE) and the Alzheimer's Disease Assessment Scale–Cognitive Subscale [ADAS-Cog; (16, 17)]; (2) the episodic memory was assessed by the immediate and delayed recall of Rey Auditory Verbal Learning Test (18); (3) the executive functions and attention were evaluated by the Trail making test (TMT) part A and B (19); (4) the language was tested by 1-min Verbal fluency test for letters (20) and 1-min Verbal fluency test for category [fruits, animals, or car trades; (20)]; and (5) planning abilities and visuospatial functions were assessed by Clock drawing and copy test (21).

In all noADMCI patients, drugs were suspended for about 24 h before rsEEG recordings. This did not ensure a complete washout of the drug for obvious ethical reasons. Instead, this procedure made it comparable rsEEG data about the drug administration in the present noADMCI patients.

All Nold subjects underwent a cognitive screening (including MMSE and GDS) as well as physical and neurological examinations to exclude any dementia or major cognitive deficit. The Nold subjects affected by any chronic systemic illnesses (e.g., diabetes mellitus) were excluded, as were the Nold subjects taking chronically psychoactive drugs. The Nold subjects with a history of previous or present neurological or psychiatric disease were also excluded. All Nold subjects had a GDS score lower than the threshold of 5 (no depression) or no depression after an interview with a physician or clinical psychologist at the time of the enrolment.

Notably, none of the present noADMCI and Nold subjects received or reported a clinical diagnosis of epilepsy or had received previous diagnosis of seizures or EEA. Specifically, in the notes on their clinical interview, none of them reported previous evidence of (1) generalized tonic–clonic seizures, myoclonic jerks, or a relevant family history of epilepsy; (2) focal seizures with or without loss of consciousness, focal motor or non-motor seizures, and focal to bilateral tonic–clonic seizures; and (3) EEA.

### rsEEG Recording

The EEG activity was recorded while the subjects were relaxed with eyes closed on a comfortable reclined chair. Instructions for rsEEG recordings encouraged the subjects to experience quiet wakefulness with muscle relaxation, no voluntary movements, no talking, and no development of systematic goal-oriented mentalization. Rather, a quiet wondering mode of mentalization was kindly required.

In the Nold subjects, rsEEG recordings lasted about 3–5 min. In the noADMCI patients, the EEG recordings were prolonged for about 30 min to increase the chance to detect EEA. No photic and hyper-ventilator stimulations were used.

The rsEEG data were recorded with a sampling frequency of 128–512 Hz and related antialiasing bandpass between 0.01 and 40–100 Hz depending on the sampling rate. Electrode montage included 19 scalp monopolar sensors placed following 10–20 System (i.e., O1, O2, P3, Pz, P4, T3, T5, T4, T6, C3, Cz, C4, F7, F3, Fz, F4, F8, Fp1, and Fp2). A frontal ground electrode was used, while cephalic or linked earlobe electrodes were used as electric references according to local methodological facilities and standards. Electrodes impedances were kept below 5 kΩ. Vertical and horizontal electro-oculographic (EOG) potentials (bandpass between 0.3 and 40–100 Hz depending on the sampling rate) were recorded to evaluate eye movements and to blink.

### Detection of Epileptiform EEG Activity (EEA)

Two expert epileptologists (C.D.B. and P.C.) of the Sapienza University of Rome visually detected artifact-free EEG data obtained in noADMCI patients from the long recordings (about 30 min) to determine the presence or absence of EEA. These epileptologists were not aware of the diagnosis of noADMCI patients. Standard bipolar longitudinal electrode montages were used for the mentioned visual analysis of EEA in the EEG traces.

During the visual analysis, the two epileptologists denoted as ***EEA*** the signal features mentioned in standard reference guidelines in Epilepsy (22–25). Specifically, the assignment of the “EEA” label fulfilled at least 4 of the following 6 criteria used for denoting the interictal epileptiform abnormalities in on-going EEG signals (25): (1) ***Di-*** or ***tri-phasic waves*** with sharp or spiky morphology (i.e., pointed peak); (2) Different ***wave-duration*** than the ongoing background activity, either shorter or longer; (3) ***Asymmetry of the waveform***: a sharply rising ascending phase and a more slowly decaying descending phase, or vice versa; (4) Transient component of EEA followed by an associated ***slow after-wave***; (5) ***Background EEG activity*** surrounding EEA disrupted by the presence of the EEA; (6) Distribution of the negative and positive EEG potentials on the scalp suggesting a ***focal source of the signal in the*** ***cortex***, corresponding to a radial, oblique, or tangential orientation of the (dipole) source.

Concerning the detection of ***spikes*** (22–24), they were defined as EEG waveforms with the following features: (1) Amplitude above the background of EEG activity; (2) A pointy shape; (3) Duration in the range from 30 to 70–80 ms; (4) Asymmetric rise-fall, and (5) A subsequent EEG slow wave (22–24). These spikes had to be characterized by relatively broad spatial distributions detectable in near scalp EEG electrodes around cortical sources and those of corresponding regions of the opposite hemisphere via callosal functional connectivity.

Compared with spikes, ***sharp waves*** were defined as EEG activities of longer duration (i.e., 70–200 ms).

Keeping in mind those guidelines (22–24), EEA was associated with EEG waveforms with the following features: focal epileptiform discharges, runs of slow spike-waves, generalized spike-waves, or spike-waves at <3 per second, giant spikes, and/or intermittent delta waveforms with spikes, sharp waves, and spike-wave discharges >3 s.

EEA was distinguished from small amounts of drowsiness-dependent temporal and frontal intermittent rhythmic delta waveforms, namely ***TIRDA*** and ***FIRDA***. Specifically, TIRDA and FIRDA were defined as delta waveforms recorded in temporal or frontal regions in (not continuous) cycles with the following features: (1) Relatively uniform morphology and duration; (2) Amplitude of 50–100 μv; and (3) No association with EEG fast activity, sharp waves or spikes, or sharply contoured activity (26).

**Figure 2:**
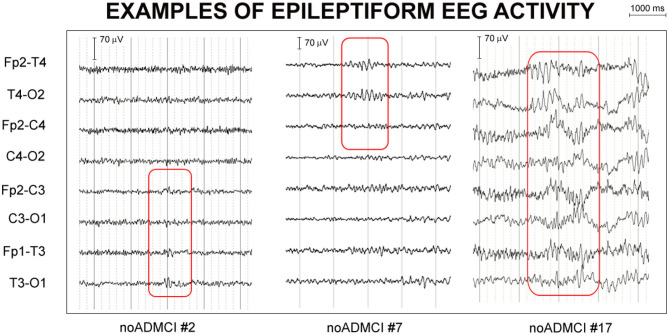
Examples of epileptiform electroencephalographic activity (EEA) in three patients with mild cognitive impairment not due to Alzheimer's disease (noADMCI) of the present study. Bipolar longitudinal electrode montages were used for the mentioned visual analysis of the electroencephalographic (EEG) traces (e.g., Fp2-T4, T4-O2, Fp2-C4, C4-O2, Fp1-T3, T3-O1, Fp1-C3, and C3-O1 electrodes of 10-20 montage system). The assignment of the “EEA” label fulfilled at least 4 of the following 6 criteria used for denoting the interictal epileptiform abnormalities in on-going EEG signals: (1) Di- or tri-phasic waves with sharp or spiky morphology; (2) Different wave-duration than the ongoing background EEG activity, either shorter or longer; (3) Asymmetry of the EEG waveform such as a sharply rising ascending phase and a more slowly decaying descending phase, or vice versa; (4) Transient component of EEA followed by an associated EEG slow after-wave; (5) Background EEG activity surrounding EEA disrupted by the presence of the EEA; (6) Distribution of the negative and positive EEG potentials on the scalp suggesting a punctual source of the signal in the cortex, corresponding to a radial, oblique or tangential orientation of the (dipole) source. The example of EEA fulfilled the criteria (1), (2), (3), and (5) in the patient noADMCI#2, (1), (2), (3), and (5) in the patient noADMCI#7, and (2), (3), (4), and (5) in the patient noADMCI#17.

For the visual detection of the mentioned EEG activity, the two epileptologists followed the next procedures: (1) sharing the rating criteria of the visual inspection; (2) review of two single cases to consolidate and harmonize the procedure; and (3) analysis of EEG signals in the remaining noADMCI patients, separately. The inter-rated congruence on EEA between the two epileptologists was >70%. In case of different judgment, the two experts discussed the case together for reaching the consensus. The consensus was always reached. Based on these criteria, they agreed that 13 out of 32 noADMCI subjects (41%) of the present study showed EEA in the long rsEEG recordings. Therefore, 19 noADMCI-noEEA (6 males; percentage of men: 31.6% male) and 13 noADMCI-EEA patients (no male) were used to test the relationship between rsEEG source activities and EEA in the present study. For illustrative purpose, Figure 2 shows examples of EEAs in the present noADMCI patients.

Table 1 reports the most relevant demographic (i.e., age, gender, and education) and clinical (i.e., MMSE, score) features of the Nold, noADMCI-noEEA, and noADMCI-EEA groups. Table 1 also reports the results of statistically significant differences among the three groups (i.e., Nold, noADMCI-noEEA, and noADMCI-EEA) for the age (ANOVA), gender (Freeman-Halton), education attainment (ANOVA), and MMSE score (Kruskal-Wallis). To consider the inflating effects of repetitive univariate tests, the statistical threshold was set at *p* < 0.0125 (i.e., 4 markers, *p* < 0.05/4 = 0.0125) to obtain the Bonferroni correction at *p* < 0.05. As expected, a statistically significant difference among the groups was found for the MMSE score (*H* = 19.1; *p* < 0.0001). Specifically, the MMSE score was higher in the Nold than the noADMCI-noEEA (*p* < 0.001) and noADMCI-EEA (*p* < 0.01) groups. On the contrary, statistically significant differences for the age, gender, and educational attainment among the three groups (i.e., Nold, noADMCI-EEA, and noADMCI-noEEA groups) were found neither considering Bonferroni correction (*p* > 0.0125) nor ignoring that correction (*p* > 0.05). Finally, statistically significant differences for the MMSE score between the two noADMCI groups (i.e., noADMCI-EEA and noADMCI-noEEA groups) were found neither considering Bonferroni correction (*p* > 0.0125) nor ignoring that correction (*p* > 0.05).

**Table 1:** Mean values (± standard error of the mean, SE) of the demographic and clinical data as well as the results of their statistical comparisons (*p* < 0.05 corrected) in the groups of healthy elderly subjects (Nold, *N* = 30), patients with mild cognitive impairment and epileptiform electroencephalographic activity not due to Alzheimer's disease (noADMCI-EEA, *N* = 13), and noADMCI without EEA (*N* = 19).

	**Nold**	**noADMCI-noEEA**	**noADMCI-EEA**	**Statistical analysis**
N	30	19	13	
Age	68.8 ± 1.2 SE	69.1 ± 1.8 SE	69.3 ± 1.8 SE	ANOVA: *p* = 0.9
Gender (M/F, %M)	6/24 (20%)	6/13 (31.6%)	0/13 (0%)	Freeman Halton test: *p* = 0.07
Education	9.8 ± 0.8 SE	9.8 ± 1.0 SE	9.0 ± 1.2 SE	ANOVA: *p* = 0.8
MMSE	28.7 ± 0.2 SE	26.2 ± 0.5 SE	26.3 ± 0.8 SE	Kruskal-Wallis ANOVA: H = 19.1, *p* < 0.00001# (Nold > noADMCI-EEA, noADMCI-noEEA)

*All ADMCI patients had all values available*.

*MMSE, Mini Mental State Evaluation; M/F, males/females*.

# *p < 0.05 corrected*.

*No significant difference for demographic data (i.e., age, gender, and education) was observed between the three groups even when a marginal threshold of p < 0.05 uncorrected was used*.

Of note, the use of selective serotonin reuptake inhibitors (SSRIs), selective serotonin and noradrenaline reuptake inhibitors (SNRIs), benzodiazepines (BZDs), non-benzodiazepines GABA acting agent (No BZDs), and acetylcholinesterase inhibitors (AChEIs) was controlled in all noADMCI patients. As mentioned above, the above 5 drugs were suspended for about 24 h before rsEEG recordings to harmonize the time of the last administration of the drug across the noADMCI patients. Practically, the patients using those drugs could take their medications immediately after EEG experiments, planned in late morning. Therefore, they just delayed the assumption of their medications for few hours in relation to their normal routine. Table 2 reports all types of the above mentioned five drugs used by the noADMCI patients. Furthermore, Table 3 reports the number and the percentages of the noADMCI-noEEA and noADMCI-EEA patients assuming the above mentioned 5 drugs before the rsEEG recordings. No statistically significant difference was found between noADMCI-noEEA and noADMCI-EEA groups in the use of the above medications even when a marginal threshold of *p* < 0.05 uncorrected was used.

**Table 2:** Type of drugs received by the noADMCI patients of the present study.

**Drugs**	**noADMCI-noEEA**	**noADMCI-EEA**
Selective serotonin reuptake inhibitors (SSRIs)	Citalopram, Escitalopram Sertraline	Citalopram, Escitalopram
Selective serotonin and noradrenaline reuptake inhibitors (SNRIs)	Venflaxine	*No drugs*
Benzodiazepine (BZD)	Alprazolam, Midazolam Lorazepam	Bromapezam, Diazepam Lorazepam
Non-benzodiazepine GABA acting agent (No BZD)	Zolpidem	*No drugs*
Acetylcholinesterase inhibitor (AChEI)	Donepezil, Rivastigmine	Donepezil

*The drugs were categorized into 5 pharmacodynamic classes: selective serotonin reuptake inhibitors (SSRIs), selective serotonin and noradrenaline reuptake inhibitors (SNRIs), benzodiazepines (BZDs), non-benzodiazepines GABA acting agent (No BZDs), and acetylcholinesterase inhibitors (AChEIs)*.

**Table 3:** Number and percentages of the noADMCI-noEEA and noADMCI-EEA patients assuming the selective serotonin reuptake inhibitors (SSRIs), selective serotonin and noradrenaline reuptake inhibitors (SNRIs), benzodiazepines (BZDs), non-benzodiazepines GABA acting agent (No BZDs), and acetylcholinesterase inhibitors (AChEIs).

**Drugs**	**noADMCI-noEEA**		**noADMCI-EEA**		**Fisher test**
	***N***	**(%)**	***N***	**(%)**	
Selective serotonin reuptake inhibitors (SSRIs)	8	42.1	6	46.2	*p* = 0.9
Selective serotonin and noradrenaline reuptake inhibitors (SNRIs)	1	5.3	0	0	*p* = 0.9
Benzodiazepine (BZD)	4	21.2	3	23.1	*p* = 0.9
Non benzodiazepine GABA acting agent (No BZD)	1	5.3	0	0	*p* = 0.9
Acetylcholinesterase inhibitor (AChEI)	2	10.5	1	0	*p* = 0.9
All drugs	10	52.6	7	53.8	*p* = 0.9

*The results of the presence or absence of statistically significant differences (Fisher test, p < 0.05 corrected) between the noADMCI-noEEA and noADMCI-EEA groups. The consumption of those drugs is also reported. All ADMCI patients had the information relative to the drug consumption available. noADMCI-noEEA, patients with mild cognitive impairment not due to Alzheimer's disease and without epileptiform EEG activity; noADMCI-EEA, patients with mild cognitive impairment not due to Alzheimer's disease and epileptiform EEG activity. No significant difference was observed between the two noADMCI groups even when a marginal threshold of p < 0.05 uncorrected was used*.

### rsEEG Preliminary Data Analysis

The preliminary analysis of the recorded rsEEG activity followed the same procedures of previous rsEEG investigations in MCI patients of our Workgroup (27–29) to make comparable the results. For this analysis, the first 3 min of the rsEEG data were divided into epochs of 2 s and analyzed offline as follows. Firstly, the rsEEG epochs with EEA, previously identified by two expert epileptologists (C.D.B. and P.C.) of the Sapienza University of Rome, were discarded. Secondly, the rsEEG epochs affected by any physiological (ocular/ blinking, muscular, and head movements) or non-physiological (sweat, bad contact between electrodes and scalp, etc.) artifacts were identified and discarded by the visual analysis of two experts of EEG signals (C.D.P., G.N., M.T.P. or R.L.). In this visual analysis, the contamination of rsEEG rhythms with the ocular activity (i.e., blinking) was evaluated in frontal electrodes (i.e., F7, F3, Fz, F4, F8, Fp1, and Fp2), comparing EOG and EEG traces. Head movement artifacts were detected by a sudden and great increase in amplitude of slow EEG waves in all scalp electrodes. Muscle tension artifacts were recognized by observing the effects of several frequency bandpass filters in different ranges and by the inspection of rsEEG power density spectra. These artifacts were reflected by unusually high and stable values of rsEEG power density from 30 to 100 Hz, which contrast with the typical declining trend of rsEEG power density from 25 Hz onward. The experimenters also detected rsEEG epochs with signs of sleep such as K complexes, sleep spindles, vertex shape waves, and slow waves. Furthermore, the two experimenters carefully rejected rsEEG epochs associated with behavioral annotations taken during the experiments (e.g., drowsiness, verbal warnings, opened eyes, arm/hand movements, etc.). To avoid residual EEA in the rsEEG epochs used for the exact Low-Resolution Brain Electromagnetic Tomography (eLORETA) source estimation, the two expert epileptologists (C.D.B. and P.C.) carefully double-checked all artifact-free rsEEG epochs used for that estimation. In all subjects, the artifact-free epochs were more than 80%.

### Spectral Analysis of rsEEG Epochs

A standard digital FFT-based analysis (Welch technique, Hanning windowing function, no phase shift) computed the power density of scalp rsEEG rhythms (0.5 Hz of frequency resolution). As mentioned above, only rsEEG epochs free from artifacts and EEA were used.

The EEG frequency bands of interest were individually identified based on the following frequency landmarks, namely the transition frequency (TF) and IAFp (30). In the EEG power density spectrum, the TF marks the transition frequency between the theta and alpha bands, defined as the minimum of the rsEEG power density between 3 and 8 Hz (between the delta and the alpha power peak). The IAF is defined as the maximum power density peak between 6 and 14 Hz. These frequency landmarks were previously well-described by Klimesch (30–32).

The TF and IAF were individually computed for each subject involved in the present study. Based on the TF and IAF, we estimated three individual frequency band ranges for each subject as follows: delta from TF−4 to TF−2 Hz, theta from TF−2 to TF Hz, and alpha from TF to IAF+2 Hz. To clarify the procedure, please consider an example of TF = 6 Hz and IAF = 10 Hz. As a result, the three frequency band ranges would be the following: delta from 2 to 4 Hz, theta from 4 to 6 Hz, and alpha from 6 to 12 Hz. Other two bands were defined based on standard fixed frequency ranges, namely beta from 14 to 30 Hz and gamma from 30 to 40 Hz. Of note, we considered a whole alpha band in line with the International Federation of Clinical Neurophysiology Glossary (33) and the Guidelines of the International Pharmaco-EEG Society (25) reporting that rsEEG alpha rhythms may have global features within a unique band. Furthermore, we used fixed frequency ranges for the beta, and gamma bands because of the individual beta and gamma frequency peaks were evident only in a few subjects (<10%). Furthermore, we selected the beginning of the beta frequency range at 14 Hz to avoid the overlapping between individual alpha and fixed beta frequency ranges (i.e., individual alpha frequency band ranged from TF to 14 Hz with an IAF = 12 Hz).

### Cortical Sources of rsEEG Epochs as Computed by eLORETA

We used the official freeware tool called exact LORETA (eLORETA) for the linear estimation of the cortical source activity generating scalp-recorded rsEEG rhythms (34). The current implementation of eLORETA uses a spherical head volume conductor model composed of the scalp, skull, and brain. In the scalp compartment, exploring electrodes can be virtually positioned to give EEG data as an input to the source estimation (34). The brain model is based on a realistic cerebral shape taken from a template typically used in the neuroimaging studies, namely that of the Montreal Neurological Institute (MNI152 template). The eLORETA freeware solves the so-called EEG inverse problem estimating “neural” current density values at any cortical voxel of the mentioned spherical head volume conductor model. The solutions are computed at all rsEEG frequency bin-by-frequency bin (0.5 Hz as frequency resolution, namely, the maximum frequency resolution allowed by the use of 2-s artifact-free EEG epochs).

The input for eLORETA source estimation is the EEG spectral power density computed at 19 scalp electrodes, placed according to the 10-20 montage system. The output is the estimation of neural currents in the brain source space formed by 6,239 voxels with 5 mm resolution, restricted to the cortical gray matter of the spherical head volume conductor model. An equivalent current dipole is located in each voxel. For each voxel, the eLORETA package provides the Talairach coordinates, the lobe, and the Brodmann area (BA).

**Figure 3:**
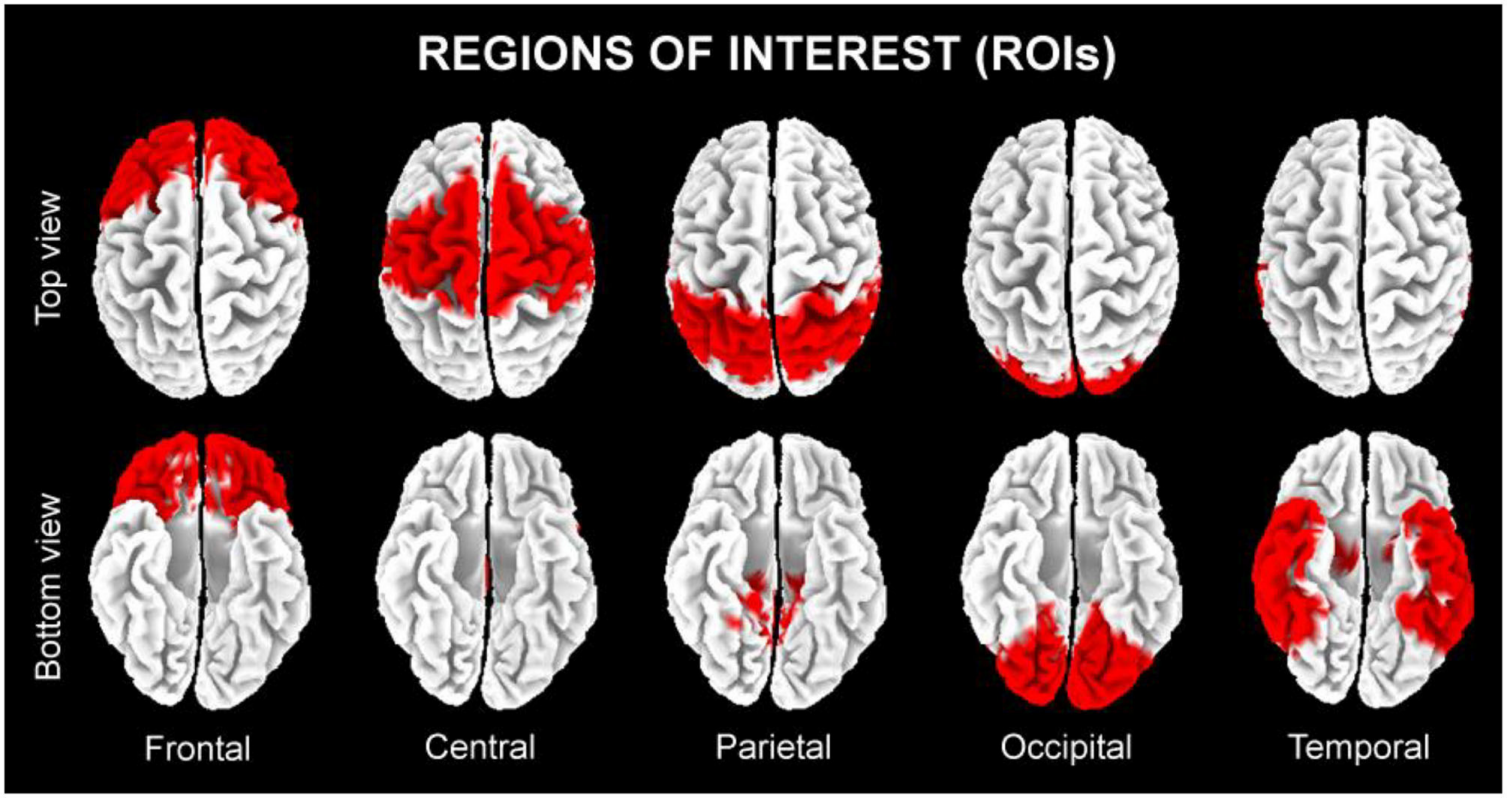
The five regions of interest (ROIs) where we averaged the solutions of the exact low-resolution brain electromagnetic tomography (eLORETA) freeware to estimate the cortical source of eyes-closed resting state electroencephalographic (rsEEG) rhythms recorded in the present seniors in the quiet wakefulness.

In line with the general low spatial resolution of the present EEG methodological approach (i.e., 19 scalp electrodes), we performed a regional analysis of the eLORETA solutions. The following five lobar macro-regions of interest (ROIs) were considered: frontal (Brodmann area, BA 8, 9, 10, 11, 44, 45, 46, and 47), central (BA 1, 2, 3, 4, and 6), parietal (BA 5, 7, 30, 39, 40, and 43), temporal (BA 20, 21, 22, 37, 38, 41, and 42), and occipital (BA 17, 18, and 19). Figure 3 shows the above mentioned five lobar ROIs. The eLORETA solution for each lobar ROI was obtained by the average of the normalized eLORETA current density values estimated at all single voxels included in that ROI. For example, the eLORETA solution for the temporal ROI was obtained by the average of the normalized eLORETA current density values estimated at all voxels included in the BA 20, 21, 22, 37, 38, 41, and 42 of the bilateral temporal lobes. Similarly, the eLORETA solution for the occipital ROI was obtained by the same principle for the BA 17, 18, and 19 of the bilateral occipital lobes.

### Statistical Analysis of the rsEEG Source Activity

The commercial tool STATISTICA 10 (StatSoft Inc., www.statsoft.com) was used to test the study hypotheses. For the statistical comparisons of the rsEEG source activities among the three groups (Nold, noADMCI-no EEA, and noADMCI-EEA), an ANOVA model was computed using the rsEEG source activities (i.e., regional normalized eLORETA solutions) as a dependent variable (*p* < 0.05). It is well-known that the use of the ANOVA models implies that dependent variables approximate Gaussian distributions, so we tested this feature in the present rsEEG source activity distributions by Kolmogorov-Smirnov test (null hypothesis of non-Gaussian distributions tested at *p* < 0.05). As the eLORETA distributions were not Gaussian in all cases, all rsEEG source activity distributions were processed by the log-10 transformation and re-tested. Such a transformation is a popular method to transform skewed data distribution with all positive values (as rsEEG source activity values are) to Gaussian distributions, thus augmenting the reliability of the ANOVA results. Indeed, the outcome of the procedure approximated all rsEEG source activity distributions to Gaussian distributions (*p* > 0.05), allowing the use of the ANOVA model.

Mauchly's test evaluated the sphericity assumption, and degrees of freedom were corrected by the Greenhouse-Geisser procedure when appropriate (*p* < 0.05). Duncan test was used for *post-hoc* comparisons (*p* < 0.05, corrected for multiple comparisons).

The results of the following statistical analyses were controlled by the iterative (leave-one-out) Grubbs' test detecting for the presence of one or more outliers in the distribution of the eLORETA source solutions. The null hypothesis of the non-outlier status was tested at the arbitrary threshold of *p* < 0.001 to remove only individual values with high probability to be outliers.

The ANOVA of the present study tested the hypothesis that rsEEG source activities (i.e., regional normalized eLORETA solutions) differed among the Nold, noADMCI-noEEA, and noADMCI-EEA groups. The ANOVA factors were Group (Nold, noADMCI-EEA, and noADMCI-noEEA), Band (delta, theta, alpha, beta, and gamma), and ROI (frontal, central, parietal, occipital, and temporal). The confirmation of the hypothesis may require: (1) a statistically significant MANOVA effect including the factor Group (*p* < 0.05) and (2) a *post-hoc* Duncan test indicating statistically significant (*p* < 0.05 Bonferroni correction for 5 frequency bands X 5 ROIs = 25, *p* < 0.05/25 = 0.002) differences in the rsEEG source activities among Nold, noADMCI-noEEA, and noADMCI-EEA groups in line with the following patterns: Nold ≠ noADMCI-EEA; Nold ≠ noADMCI-noEEA; and noADMCI-EEA ≠ noADMCI-noEEA (*p* < 0.002 = *p* < 0.05 corrected).

### Discriminant Analysis in the noADMCI Patients

To minimize the statistical analyses and false discoveries in the present study, only regional normalized eLORETA solutions showing statistically significant differences (*p* < 0.05) between the noADMCI-EEA and noADMCI-noEEA groups (i.e., effects of the factor Group and Duncan *post-hoc*) in the MANOVA design were used as variables of interest for discriminant analyses in the noADMCI-EEA and noADMCI-noEEA individuals.

For the discriminant analysis, the classifications were performed by GraphPad Prism software (GraphPad Software, Inc, California, USA) using its implementation of Receiver Operating Characteristic (ROC) curves (35). The following indexes measured the results of the binary classifications in ROC curve analysis: (1) sensitivity; it measures the rate of the cases who were correctly classified as cases; (2) specificity; it measures the rate of the controls who were correctly classified as controls; (3) accuracy; it is the mean between the sensitivity and specificity weighted for the number of cases and controls (4) positive predictive value; probability that the disease is present when the test is positive; (5) negative predictive value; the probability that the disease is not present when the test is negative; and (6) the AUROC curve. For the sake of brevity, the AUROC curve was used as a major reference index of the global classification accuracy.

## Results

### Statistical Comparison of Clinical, Anthropometric, Genetic, Cerebrospinal Fluid, and Neuropsychological Markers in the noADMCI-noEEA and noADMCI-EEA Groups

Table 4 reports the most relevant clinical (i.e., Geriatric Depression Scale, GDS, Clinical Dementia Rating, CDR, and Hachinski Ischemic Score, HIS), genetic (i.e., APOE genotyping), and cerebrospinal fluid (i.e., Aβ42, t-tau, p-tau, Aβ42/p-tau) features of the noADMCI-noEEA and noADMCI-EEA groups. Table 4 also reports the results of the presence or absence of statistically significant differences (*p* < 0.05) between the two groups (i.e., noADMCI-noEEA and noADMCI-EEA) for the above mentioned clinical (*T*-test), genetic (Fisher test), and cerebrospinal fluid (*T*-test) markers. To consider the inflating effects of repetitive univariate tests, the statistical threshold was set at *p* < 0.006 (i.e., 8 markers, *p* < 0.05/8 = 0.00625) to obtain the Bonferroni correction at *p* < 0.05. Statistically significant differences were found neither considering that correction (*p* > 0.00625) nor ignoring that correction (*p* > 0.05).

**Table 4:** Mean values (±SE) of the clinical (i.e., Geriatric Depression Scale, Clinical Dementia Rating, and Hachinski Ischemic Score) genetic (i.e., Apolipoprotein E genotyping, APOE), and cerebrospinal fluid (i.e., beta amyloid 1-42, Aβ 42; protein tau, t-tau; and phosphorylated form of protein tau, p-tau) data as the results of their statistical comparisons (*p* < 0.05 corrected) in the groups of noADMCI-noEEA (*N* = 19) and noADMCI-EEA (*N* = 13) patients.

	**noADMCI-noEEA**	**noADMCI-EEA**	**Statistical analyses**
**Clinical, genetic (APOE) and cerebrospinal fluid markers in noADMCI-noEEA and noADMCI-EEA**			
**CLINICAL MARKERS**			
Geriatric depression scale (GDS)	2.3 ± 0.5	2.3 ± 0.6	*T*-test: *p* = 0.9
Clinical dementia rating (CDR)	0.5 ± 0.0	0.5 ± 0.0	*T*-test: *p* = 1.0
Hachinski ischemic score (HIS)	1.2 ± 0.3	1.2 ± 0.2	*T*-test: *p* = 0.9
**GENETIC MARKER**			
APOE4 (%)	10.5%	7.7%	Fisher test: *p* = 0.9
**CEREBROSPINAL FLUID MARKERS (pg/mL)**			
Aβ42	1,000 ± 44	1,087 ± 73	*T*-test: *p* = 0.25
p-tau	43 ± 2	51 ± 5	*T*-test: *p* = 0.15
t-tau	276 ± 29	343 ± 48	*T*-test: *p* = 0.25
Aβ42/p-tau	24 ± 1	23 ± 2	*T*-test: *p* = 0.6

*All ADMCI patients had all values of those markers available. In line with the inclusion criteria, all noADMCI patients had CDR score of 0.5, GDS score ≤ 5, HIS score ≤ 4, and Aβ 42 level in the cerebrospinal fluid (CSF) > 550 pg/mL*.

*noADMCI-noEEA, patients with mild cognitive impairment not due to Alzheimer's disease and without epileptiform EEG activity; noADMCI-EEA, patients with mild cognitive impairment not due to Alzheimer's disease and epileptiform EEG activity. No significant difference was observed between the two noADMCI groups even when a marginal threshold of p < 0.05 uncorrected was used*.

Table 5 reports the mean values (± SE) of the anthropometric features (i.e., weight, height, and body mass index) and cardiocirculatory markers (i.e., systolic pressure, diastolic pressure, pulse pressure, mean arterial pressure, and heart frequency) measured in the noADMCI-noEEA and noADMCI-EEA groups. Table 5 also reports the results of the presence or absence of statistically significant differences (*T*-test; log-10 transformed data) between the two groups (i.e., noADMCI-noEEA and noADMCI-EEA) for the above markers used. To consider the inflating effects of repetitive univariate tests, the statistical threshold was set at *p* < 0.006 (i.e., 8 markers, *p* < 0.05/8 = 0.00625) to obtain the Bonferroni correction at *p* < 0.05. No statistically significant difference was found (*p* > 0.05 corrected). However, the mean height was significantly greater in the noADMCI-noEEA as compared to the noADMCI-EEA group with an explorative statistical threshold of *p* < 0.05 uncorrected.

**Table 5:** Mean values (± SE) of the anthropometric features (i.e., weight, height, and body mass index) and cardiocirculatory markers (i.e., systolic pressure, diastolic pressure, pulse pressure, mean arterial pressure, and heart frequency) as well as the results of their statistical comparisons (*T*-test on log-10 transformed data; *p* < 0.05 corrected) in the groups of noADMCI-noEEA (*N* = 19) and noADMCI-EEA (*N* = 13) patients.

	**noADMCI-noEEA**	**noADMCI-EEA**	***T*-test**
**Anthropometric markers, blood pressure and pulse in noADMCI-noEEA and noADMCI-EEA**			
Weight (kg)	72 ± 4 (*N* = 19)	71 ± 4 (*N* = 12)	*p* = 0.4
Height (cm)	163 ± 3 (*N* = 19)	157 ± 2 (*N* = 12)	*p* = 0.03
Body mass index (BMI)	26.9 ± 0.9 (*N* = 19)	29.1 ± 1.8 (*N* = 12)	*p* = 0.1
Systolic pressure (mmHg)	122± 8 (*N* = 18)	135 ± 4 (*N* = 11)	*p* = 0.2
Diastolic pressure (mmHg)	73 ± 3 (*N* = 18)	76 ± 4 (*N* = 11)	*p* = 0.3
Pulse pressure (mmHg)	55 ± 4 (*N* = 18)	59 ± 5 (*N* = 11)	*p* = 0.2
Mean arterial pressure (MAP, mmHg)	92 ± 3 (*N* = 18)	95 ± 3 (*N* = 11)	*p* = 0.2
Heart frequency/min	67 ± 3 (*N* = 18)	66 ± 3 (*N* = 11)	*p* = 0.4

*The table also reports the number of participants for which the values of those markers were available*.

*noADMCI-noEEA, patients with mild cognitive impairment not due to Alzheimer's disease and without epileptiform EEG activity; noADMCI-EEA, patients with mild cognitive impairment not due to Alzheimer's disease and epileptiform EEG activity. A marginal difference was observed between the two noADMCI groups when a marginal threshold of p < 0.05 uncorrected was used. Namely the height was higher in the noADMCI-no EEA than the noADMCI-EEA group*.

Table 6 reports the mean values (± SE) of the following neuropsychological tests in the noADMCI-noEEA and noADMCI-EEA groups: ADAS-Cog, Rey Auditory Verbal Learning Test (immediate and delayed recall), TMT B-A, Verbal fluency for letters, Verbal fluency for the category, Clock drawing, and Clock copy. Table 6 also includes the cut-off scores of above-mentioned neuropsychological tests (20, 36–39) and the results of the presence or absence of statistically significant differences (*T*-test; log-10 transformed data) between the two groups (i.e., noADMCI-noEEA and noADMCI-EEA) for the neuropsychological tests used. To consider the inflating effects of repetitive univariate tests, the statistical threshold was set at *p* < 0.006 (i.e., 8 neuropsychological tests, *p* < 0.05/8 = 0.00625) to obtain the Bonferroni correction at *p* < 0.05. No statistically significant difference was found (*p* > 0.05 corrected). However, the following results were observed using an explorative statistical threshold of *p* < 0.05 uncorrected: (1) A worsening of the TMT B-A score in the noADMCI-noEEA as compared to the noADMCI-EEA group (*p* = 0.03) and (2) A worsening of the Clock copy score in the noADMCI-EEA as compared to the noADMCI-noEEA group (*p* = 0.03), thus showing variable deficits in cognitive domains in the two groups.

**Table 6:** Mean values (± SE) of the neuropsychological scores (i.e., ADAS-Cog, Rey Auditory Verbal Learning Test immediate recall, Rey Auditory Verbal Learning Test delayed recall, Trail Making Test part B-A, Verbal fluency for letters, Verbal fluency for category, Clock drawing, and Clock copy) as well as the results of their statistical comparisons (*T*-test on log-10 transformed data; *p* < 0.05 corrected) in the groups of noADMCI-noEEA (*N* = 19) and noADMCI-EEA (*N* = 13) patients.

		**noADMCI-noEEA**	**noADMCI-EEA**	
	**Cut-off of abnormality**	**Mean ± SE** **(%subjects with abnormal score)**	**Mean ± SE** **(%subjects with abnormal score)**	***T*-test**
**Neuropsychological markers in noADMCI-noEEA and noADMCI-EEA**				
ADAS-Cog	*≥ 17*	19.7 ± 1.5 *(63.2%) N* = 19	17.9 ± 1.4 *(51%) N* = 12	*p* = 0.2
RAVLT, immediate recall	*< 28.53*	31.8 ± 2.1 *(42.1%) N* = 19	34.5 ± 1.7 *(15.4%) N* = 13	*p* = 0.1
RAVLT, delayed recall	* < 4.69*	4.1 ± 0.7*(57.9%) N* = 19	5.1 ± 0.8*(41.7%) N* = 12	*p* = 0.2
Trail Making test B-A	*≥ 187*	130.1 ± 17.9 *(25%) N* = 16	80.1 ± 15.1 *(9.1%) N* = 11	*p* = 0.03
Clock drawing	*> 3*	4.1 ± 0.2 *(73.7%) N* = 19	4.1 ± 0.3 *(76.9%) N* = 13	*p* = 0.4
Clock copy	*> 3*	4.5 ± 0.2 *(89.5%) N* = 19	4.9 ± 0.1 *(100%) N* = 13	*p* = 0.03
Letter fluency	* < 17*	24.9 ± 3.1 *(36.8%) N* = 19	31.5 ± 3.1 *(7.7%) N* = 13	*p* = 0.06
Letter category	* < 25*	31.4 ± 3.8 *(36.8%) N* = 19	33.9 ± 3.7 *(15.4%) N* = 13	*p* = 0.2

*The cut-off scores of the neuropsychological tests are also reported. The table also reports the number of participants for which the neuropsychological tests were available. Legend: noADMCI-noEEA, patients with mild cognitive impairment not due to Alzheimer's disease and without epileptiform EEG activity; noADMCI-EEA, patients with mild cognitive impairment not due to Alzheimer's disease and epileptiform EEG activity; ADAS-Cog, Alzheimer's Disease Assessment Scale-Cognitive Subscale; RAVLT, Rey Auditory Verbal Learning Test; TMT B-A, Trail Making Test part B-A. A marginal difference was observed between the two noADMCI groups when a marginal threshold of p < 0.05 uncorrected was used. Namely the trail making A-B score was worse in the noADMCI-noEEA than the noADMCI-EEA group, while the clock test score was worse in the noADMCI-EEA than the noADMCI-no EEA group (p = 0.03 uncorrected)*.

### Statistical Comparison of the rsEEG Source Activities in the Nold, noADMCI-EEA, and noADMCI-noEEA Groups

Table 7 reports the mean values of TF and IAF in the Nold, noADMCI-EEA, and noADMCI-noEEA groups, together with the results of the statistical comparisons among them (ANOVA, *p* < 0.05). The mean TF was of 5.6 Hz (± 0.2 standard error mean, SE) in the Nold group, 5.6 Hz (± 0.2 SE) in the noADMCI-noEEA group, and 5.2 Hz (± 0.2 SE) in the noADMCI-EEA group. Furthermore, the mean IAF was of 9.6 Hz (± 0.2 SE) in the Nold group, 9.4 Hz (± 0.3 SE) in the noADMCI-noEEA group, and 9.0 Hz (± 0.3 SE) in the noADMCI-EEA group. To consider the inflating effects of repetitive univariate tests, the statistical threshold was set at *p* < 0.025 (i.e., 2 markers, *p* < 0.05/2 = 0.025) to obtain the Bonferroni correction at *p* < 0.05. Statistically significant differences were found neither considering that correction (*p* > 0.025) nor ignoring that correction (*p* > 0.05).

**Table 7:** Mean values (± SE) of transition frequency (TF) and individual alpha frequency peak (IAF) computed from resting state EEG (rsEEG) power density spectra as well as the results of their statistical comparisons (ANOVA; *p* < 0.05 corrected) in the Nold (*N* = 30), noADMCI-noEEA (*N* = 19), and noADMCI-EEA (*N* = 13) groups.

	**Nold**	**noADMCI-noEEA**	**noADMCI- EEA**	**Statistical analysis**
TF (Hz)	5.6 ± 0.2 SE	5.6 ± 0.2 SE	5.2 ± 0.2 SE	ANOVA: *p* = 0.2
IAF (Hz)	9.6 ± 0.2 SE	9.4 ± 0.3 SE	9.0 ± 0.2 SE	ANOVA: *p* = 0.3

*noADMCI-noEEA, patients with mild cognitive impairment not due to Alzheimer's disease and without epileptiform EEG activity; noADMCI-EEA, patients with mild cognitive impairment not due to Alzheimer's disease and epileptiform EEG activity. No significant difference for TF and IAF was observed among the three groups even when a marginal threshold of p < 0.05 uncorrected was used*.

**Figure 4:**
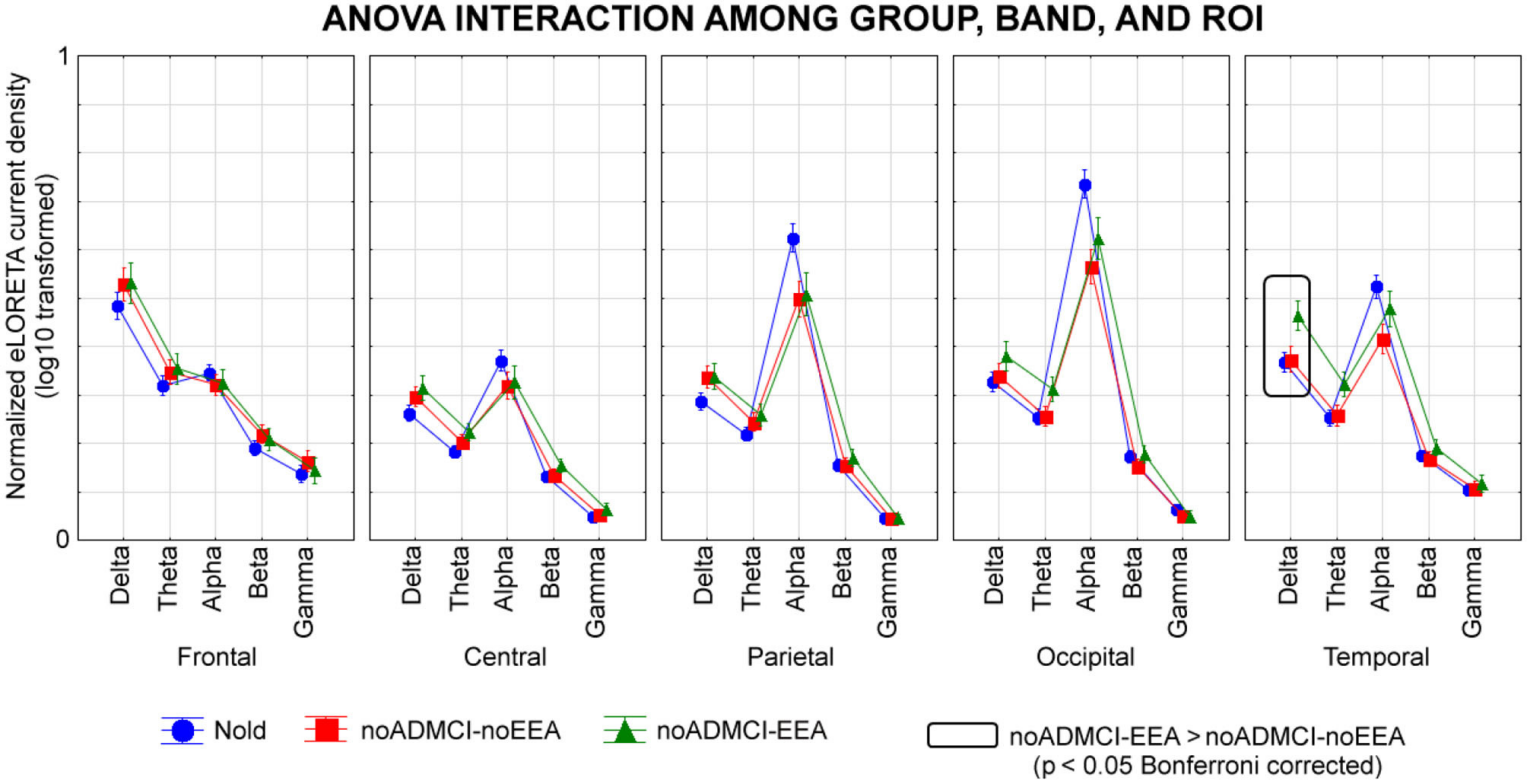
Regional normalized exact low-resolution brain electromagnetic tomography (eLORETA) solutions (mean across subjects, log-10 transformed) modeling cortical sources of eyes-closed resting state electroencephalographic (rsEEG) rhythms relative to a statistical ANOVA interaction among the factors Group (healthy elderly subjects, Nold, *N* = 30; = patients with mild cognitive impairment not due to Alzheimer's disease and without epileptiform EEG activity, noADMCI-noEEA, *N* = 19; patients with mild cognitive impairment not due to Alzheimer's disease and epileptiform EEG activity, noADMCI-EEA, *N* = 13), Band (delta, theta, alpha, beta, and gamma), and Region of interest, ROI (central, frontal, parietal, occipital, and temporal). This ANOVA design used the regional normalized eLORETA solutions as a dependent variable. Regional normalized eLORETA solutions modeled the rsEEG relative power spectra as revealed by a sort of “virtual” intracranial macro-electrodes located on the macro-cortical regions of interest. Legend: the rectangles indicate the cortical regions and frequency bands in which the eLORETA solutions statistically presented a significant eLORETA pattern noADMCI-no noEEA ≠ noADMCI-noEEA (*p* < 0.05 corrected = *p* < 0.002).

Figure 4 shows the mean values (± SE, log-10 transformed) of regional rsEEG source activities (i.e., normalized eLORETA solutions) relative to a statistically significant ANOVA interaction effect (*F* = 1.5; *p* < 0.05) among the factors Group (Nold, noADMCI-noEEA, noADMCI-EEA), Band (delta, theta, alpha, beta, and gamma), and ROI (frontal, central, parietal, occipital, and temporal). Compared with the Nold group, the noADMCI-noEEA and noADMCI-EEA groups showed a substantial decrease of the eLORETA solutions in posterior alpha sources. Furthermore, compared with the Nold group, the noADMCI-EEA groups showed a substantial increase of the eLORETA solutions in widespread delta sources. Finally, delta eLORETA solutions also showed a substantial increase in temporal delta sources in the noADMCI-EEA than the noADMCI-noEEA group.

The Duncan planned *post-hoc* testing (threshold at *p* < 0.05 corrected) showed the following significant effects in relation to the Nold group: (1) lower alpha source activities in the parietal (*p* = 0.000005), occipital (*p* = 0.000005), and temporal (*p* = 0.00001) regions in the noADMCI-noEEA group (*p* < 0.001); (2) lower alpha source activities in the parietal (*p* = 0.00005) and occipital (*p* = 0.00001) regions in the noADMCI-EEA group; and (3) higher delta source activities in the temporal region (*p* = 0.00005) in the in the noADMCI-EEA group. Furthermore, there was a statistical trend for greater delta source activities in the central, parietal, and occipital regions in the noADMCI-group (*p* < 0.05 uncorrected).

The Duncan planned *post-hoc* testing (threshold at *p* < 0.05 corrected) between the two noADMCI groups showed greater delta source activities in the temporal region in the noADMCI-EEA than the noADMCI-noEEA group (*p* = 0.0001). The above findings were not due to outliers from those individual eLORETA solutions (log-10 transformed), as shown by Grubbs' test with an arbitrary threshold of *p* < 0.001.

### Discriminant Analyses in the noADMCI Patients

**Figure 5:**
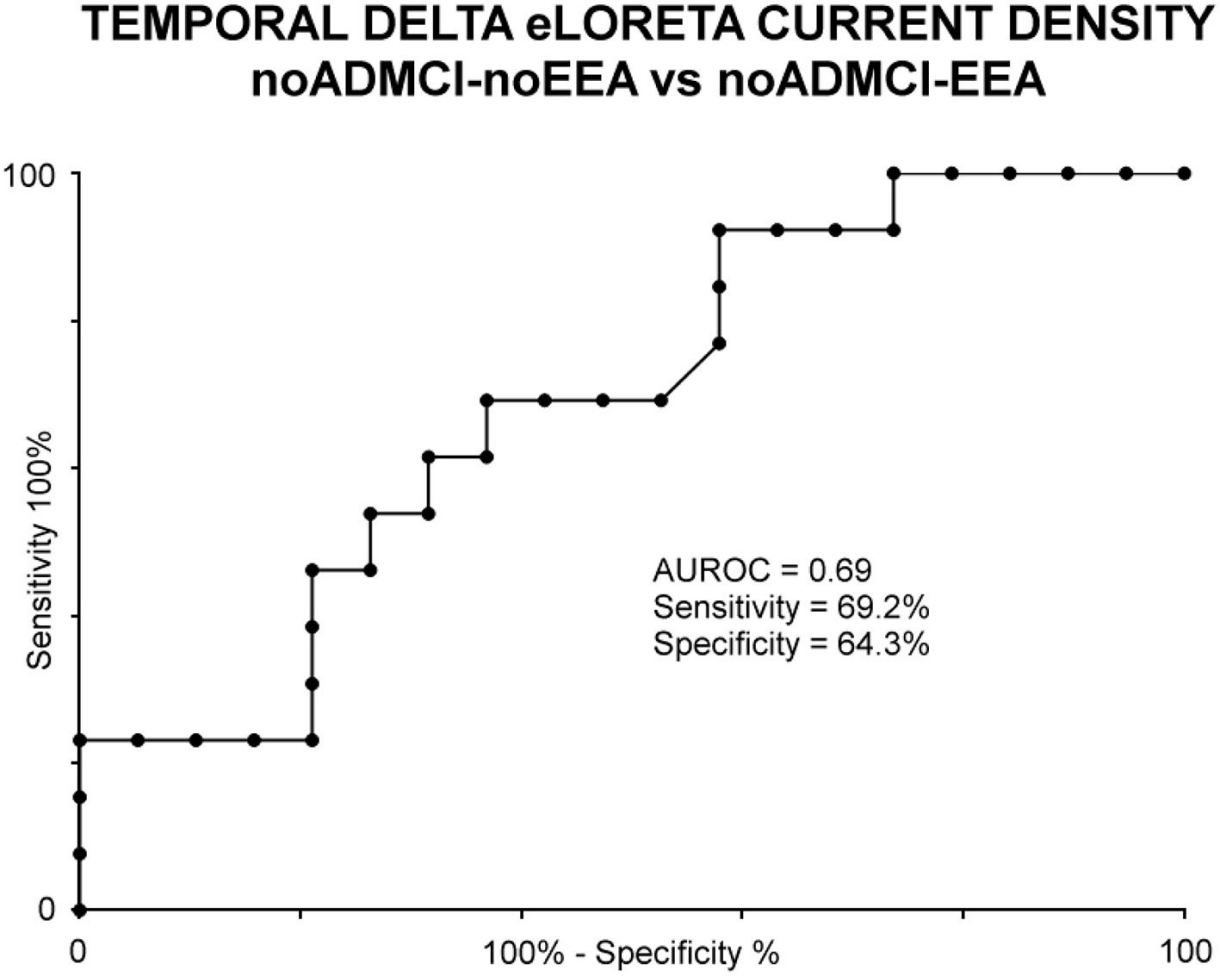
Receiver operating characteristic (ROC) curve illustrating the classification of the noADMCI-noEEA (*N* = 19) and noADMCI-EEA (*N* = 13) individuals based on regional normalized eLORETA solutions (log-10 transformed) modeling temporal delta sources. The area under the receiving operator characteristic (AUROC) curve showed moderate classification accuracies.

As an exploratory analysis at the individual level, the temporal delta source activity (i.e., regional normalized eLORETA solutions, log-10 transformed) showing statistically significant differences between the noADMCI-noEEA and noADMCI-EEA groups (*p* < 0.05 corrected) were used as an input for the computation of the AUROC curves. The AUROC computation tested the ability of that regional normalized eLORETA solution in the classification between noADMCI-noEEA and noADMCI-EEA individuals. Results showed the following moderate classification accuracy in the discrimination of individuals of the two groups: a sensitivity of 69.2%, a specificity of 64.3%, an accuracy of 65.7%, a positive predictive value of 39.2%, a negative predictive value of 86.2%, and an AUROC curve of 0.69. Figure 5 plots the scatterplots of that effect.

### Control Analyses

**Figure 6:**
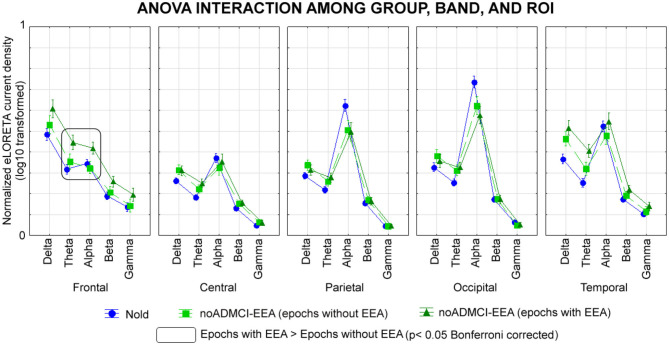
Regional normalized eLORETA solutions (mean across subjects, log-10 transformed) of cortical sources of rsEEG rhythms relative to a statistical ANOVA interaction among the factors Group (Nold, N= 30; noADMCI-EEA using the rsEEG epochs without EEA, *N* = 13; noADMCI-EEA using the rsEEG epochs with EEA, *N* = 13), Band (delta, theta, alpha, beta, and gamma), and ROI (central, frontal, parietal, occipital, and temporal). This ANOVA design used the regional normalized eLORETA solutions as a dependent variable. Legend: the rectangles indicate the cortical regions and frequency bands in which the eLORETA solutions statistically presented a significant eLORETA pattern noADMCI-EEA using the rsEEG epochs without EEA ≠ noADMCI-EEA using the rsEEG epochs with EEA (*p* < 0.05 corrected = *p* < 0.002).

A first control analysis was performed to confirm that the above rsEEG (eLORETA) source differences between the noADMCI-noEEA and noADMCI-EEA groups were not due to residual EEA in the artifact-free rsEEG epochs of the noADMCI-EEA group. Specifically, we evaluated whether the rsEEG source activities (i.e., regional normalized eLORETA solutions) may differ in the rsEEG epochs with vs. without EEA in the noADMCI-EEA group. For each noADMCI-EEA patient, the procedure was performed as follows: (1) the eLORETA freeware estimated the rsEEG cortical sources in the rsEEG epochs with EEA (see *Cortical sources of rsEEG epochs as computed by eLORETA* section in Material and Methods); (2) for each frequency band of interest and ROI, the rsEEG source activities (i.e., regional normalized eLORETA solutions) were log-10 transformed to make them Gaussian before the subsequent parametric statistical analysis; (3) an ANOVA was computed using the rsEEG source activities (i.e., regional normalized eLORETA solutions) as a dependent variable (*p* < 0.05). The ANOVA factors were Group (Nold, noADMCI-EEA using the rsEEG epochs without EEA, and noADMCI-EEA using the rsEEG epochs with EEA), Band (delta, theta, alpha, beta, and gamma), and ROI (frontal, central, parietal, occipital, and temporal). The results showed a statistically significant interaction effect (*F* = 1.9; *p* < 0.001; see Figure 6) among the three factors. The Duncan planned *post-hoc* testing (*p* < 0.05 Bonferroni corrected for 5 frequency bands X 5 ROIs = 25, *p* < 0.05/25 = 0.002) unveiled that the discriminant pattern noADMCI-EEA using the rsEEG epochs without EEA < noADMCI-EEA using the rsEEG epochs with EEA was fitted by the frontal theta (*p* = 0.0005) and frontal alpha (*p* = 0.0005). Notably, the significant effect of the above ANOVA was controlled by the iterative (leave-one-out) Grubbs' test detecting for the presence of one or more outliers in the distribution of the regional normalized eLORETA solutions (*p* < 0.001). No outlier was found (*p* > 0.001). Overall, that control analysis showed that: (1) the rsEEG source activities (i.e., regional normalized eLORETA solutions) differed in the rsEEG epochs with vs. without EEA in the noADMCI-EEA group; (2) the cortical sources showing differences between the rsEEG epochs with vs. without EEA in the noADMCI-EEA group (i.e., frontal theta and frontal alpha) were characterized by different spatial-frequency features as compared to those showing differences between the noADMCI-EEA and noADMCI-noEEA groups (i.e., temporal delta). Those findings confirm that rsEEG (eLORETA) source differences between the noADMCI-noEEA and noADMCI-EEA groups may not depend on residual EEA in the artifact-free EEG epochs used for the eLORETA source estimation.

Furthermore, a second control analysis was performed to confirm that the above rsEEG (eLORETA) source differences between the noADMCI-noEEA and noADMCI-EEA groups may not be due to (1) the global neurodegeneration of cerebral cortex; (2) the neurodegeneration of cortical structures as the mesial temporal cortex, basal ganglia, and lateral ventricle; and (3) cerebrovascular lesions. In that analysis, we evaluated whether MRI markers may differ between the noADMCI-noEEA and noADMCI-EEA groups. For each noADMCI patient, the procedure was as follows: (1) the MRI markers included: (i) the total gray matter (GM) and white matter (WM) volumes (normalized respect to total intracranial volume) and the total cortical thickness; (ii) the volumes of caudate, putamen, pallidum, accumbens, hippocampus, amygdala, and lateral ventricle (normalized with respect to total intracranial volume), and the cortical thicknesses of entorhinal cortex; and (iii) the WM hypointensity and WM lesions; (2) the MRI markers were log-10 transformed to make them Gaussian before the subsequent parametric statistical analysis; (3) *T*-tests were computed to evaluate the presence or absence of statistically significant differences between the two noADMCI groups for the MRI markers mentioned above. To consider the inflating effects of repetitive univariate tests, the statistical threshold was set at *p* < 0.0038 (i.e., 13 MRI markers, *p* < 0.05/13 = 0.0038) to obtain the Bonferroni correction at *p* < 0.05 corrected. Statistically significant differences were found neither considering that correction (*p* > 0.0038) nor ignoring that correction (*p* > 0.05; see Table 8). Overall, that control analysis confirmed that the rsEEG (eLORETA) source differences between the noADMCI-noEEA and noADMCI-EEA groups were not merely due to the neurodegeneration of cortical structures or cerebrovascular lesions.

**Table 8:** Mean values (± SE) of the magnetic resonance imaging (MRI) markers (i.e., volumes of the total gray matter, total white matter, caudate, putamen, pallidum, accumbens, hippocampus, amygdale, and lateral ventricle; cortical thicknesses of the total cortex and entorhinal cortex; white matter hypointensity and lesions) as well as the results of their statistical comparisons (*T*-test on log-10 transformed data; *p* < 0.05 corrected) in the groups of noADMCI-noEEA (*N* = 19) and noADMCI-EEA (*N* = 13) patients.

	**noADMCI-noEEA**	**noADMCI-EEA**	***T*-test**
**MRI markers in noADMCI-noEEA and noADMCI-EEA**			
**GLOBAL MARKERS**			
Normalized total WM volume	0.28 ± 0.01	0.30 ± 0.01	*p* = 0.1
Normalized total GM volume	0.40 ± 0.01	0.40 ± 0.01	*p* = 0.4
Cortical thickness	4.9 ± 0.1	4.8 ± 0.1	*p* = 0.1
**BASAL GANGLIA MARKERS**			
Normalized caudate volume	0.0050 ± 0.0003	0.0047 ± 0.0001	*p* = 0.1
Normalized putamen volume	0.0061 ± 0.0002	0.0060 ± 0.0003	*p* = 0.4
Normalized pallidum volume	0.0024 ± 0.0001	0.0026 ± 0.0001	*p* = 0.1
Normalized accumbens volume	0.00058 ± 0.00004	0.00065 ± 0.00004	*p* = 0.1
**MESIAL TEMPORAL MARKERS**			
Normalized hippocampus volume	0.0051 ± 0.0002	0.0051 ± 0.0002	*p* = 0.1
Normalized amygdale volume	0.0019 ± 0.0001	0.0020 ± 0.0001	*p* = 0.3
Entorhinal cortical thickness	6.7 ± 0.2	6.5 ± 0.3	*p* = 0.3
**VENTRICULAR MARKERS**			
Normalized lateral ventricle volume	0.022 ± 0.003	0.018 ± 0.003	*p* = 0.1
Hypo-intensity/lesion WM markers			
WM hypo-intensity	3,251 ± 515	2,310 ± 397	*p* = 0.1
WM lesions	2,541 ± 790	2,869 ± 959	*p* = 04

*The volumes were normalized with reference to the total intracranial volume. All ADMCI patients had all values of those MRI markers available*.

*noADMCI-noEEA, patients with mild cognitive impairment not due to Alzheimer's disease and without epileptiform EEG activity; noADMCI-EEA, patients with mild cognitive impairment not due to Alzheimer's disease and epileptiform EEG activity. No significant difference was observed between the two noADMCI groups even when a marginal threshold of p < 0.05 uncorrected was used*.

Finally, a third control analysis tested whether the results of the main statistical analysis may be affected by the slight but no significant difference of the gender distribution among the three groups. To this aim, we performed an ANOVA using the gender as a covariate. In that ANOVA, the regional normalized eLORETA solutions were used as a dependent variable and Group (Nold, noADMCI-EEA, and noADMCI-noEEA), Band (delta, theta, alpha, beta, and gamma), and ROI (frontal, central, parietal, occipital, and temporal) as factors. Bonferroni correction was applied for 5 frequency bands X 5 ROIs, *p* < 0.05/25 = 0.002. The ANOVA showed a statistically significant interaction effect (*F* = 1.4; *p* < 0.05) among the factors Group, Band, and ROI. The Duncan planned *post-hoc* testing (threshold at *p* < 0.05 corrected) showed the following significant effects in relation to the Nold group: (1) lower alpha source activities in the parietal (*p* = 0.00001), occipital (*p* = 0.000005), and temporal (*p* = 0.00001) regions in the noADMCI-noEEA; (2) lower alpha source activities in the parietal (*p* = 0.00001) and occipital (*p* = 0.00001) regions in the noADMCI-EEA group; and (3) higher delta source activities in the temporal region (*p* = 0.0001) in the in the noADMCI-EEA group. The Duncan planned *post-hoc* testing (threshold at *p* < 0.05 corrected) between the two noADMCI groups showed greater delta source activities in the temporal region in the noADMCI-EEA than the noADMCI-noEEA group (*p* = 0.0001). Those findings confirm that rsEEG (eLORETA) source differences among the three groups may not depend on the difference in gender distribution.

## Discussion

In recent unpublished data of our research group, we showed that 25% of ADMCI patients with no clinical diagnosis of epilepsy or previous report of seizures/epileptiform EEG patterns were associated with epileptiform EEG activity and related abnormalities in widespread (i.e., central, parietal, occipital, and temporal) delta and temporal theta rsEEG source activities compared with Nold subjects and control ADMCI patients without epileptiform EEG activity. In this explorative and retrospective study, we used the same methodology to evaluate whether a similar association between epileptiform EEG activity and rsEEG source abnormalities was observable even in amnesic MCI patients unaffected by clinical epilepsy and AD (noADMCI) based on standard diagnostic biomarkers (13).

As a first novel result of the present study, we showed epileptiform EEG activity in about 41% of 32 noADMCI individuals, thus suggesting that regardless the disease etiology, a substantial percentage of amnesic MCI patients unaffected by clinical epilepsy and AD may show that epileptiform EEG activity. These findings complement investigations of independent research groups using different modalities of EEG recordings (3, 40). Those previous studies reported significant percentages of epileptiform EEG activity (≥40%) in AD patients with cognitive deficits by the following data acquisition protocols: (1) the collection of whole-head MEG activity in the resting state condition, which is sensitive to epileptiform sources localized in fissures of temporal lobes (7); (2) intracranial EEG recordings from mesial temporal regions by electrodes inserted through the foramen ovale of the head (41); and (3) 24-h scalp EEG recordings allowing the detection of epileptiform EEG activity in both wakefulness and sleep (5). Compared with those procedures, the present methodology was effective in noADMCI patients and presents interesting features in terms of low costs, non-invasiveness, moderate human efforts, and high feasibility and large availability in the middle- and low-income countries in the perspective of both clinical practice and multi-centric clinical trials.

At the group level, both noADMCI-noEEA and noADMCI-EEA patients were characterized by abnormalities in cortical sources of rsEEG rhythms at the alpha frequency band. Specifically, posterior alpha source activities, which are typically dominant in eyes-closed resting state condition in healthy adults, showed a substantial decrease in magnitude in both noADMCI groups as compared to the Nold group. These findings agree with previous evidence of our research Consortium obtained in patients with MCI due to AD, Parkinson's (PDMCI), and Lewy Body disease (LBDMCI) using the same general methodology (27–29).

As a second novel result of the present study, we reported that at the group level, the epileptiform EEG activity in the noADMCI patients was associated with neither peculiar abnormality in the IAF peak nor alpha source activities in the noADMCI-EEA patients. This result suggests that epileptiform EEG activity may not affect dominant posterior alpha rhythms in noADMCI patients. In contrast, we observed greater temporal delta source activities in the noADMCI group with epileptiform EEG activity as compared to the groups of Nold subjects and noADMCI patients without epileptiform EEG activity. At the individual level, that source activity reached the accuracy (e.g., AUROC curve) of 69% in the classification between the noADMCI-EEA and noADMCI-noEEA individuals. This relatively poor classification accuracy was probably due to the etiological heterogeneity of the enrolled noADMCI patients and related rsEEG source activity.

Of note, the present findings did not depend on differences between the two noADMCI groups in several relevant features typically associated to main dementing factors: (1) demography, (2) clinical status (e.g., GDS, CDR, HIS); (3) APOE genotyping, (4) amyloidosis or tauopathy (e.g., amyloid beta 1-42, protein tau and phosphorylated form of tau levels in the CSF); (5) cardiocirculatory condition (e.g., systolic pressure, diastolic pressure, pulse pressure, mean arterial pressure, and heart frequency); (6) anthropometric features (i.e., body mass index); and (7) neurodegeneration (e.g., cortical atrophy of total gray matter, total white matter, mesial temporal cortex, basal ganglia, lateral ventricle). Neither the present epileptiform EEG activity in the noADMCI patients may depend on differences in the global cognitive status (e.g., MMSE and ADAScog scores), episodic memory (e.g., Rey Auditory Verbal Learning Test Immediate and delayed recall scores), and language (e.g., Verbal fluency test for letters and category scores) with 2 exceptions. There were more abnormal (1) planning abilities and visuospatial functions (e.g., Clock copy score) in the noADMCI with than without epileptiform EEG activity and (2) executive functions (e.g., Trail Making Test B-A score) in the noADMCI without than with epileptiform EEG activity (*p* < 0.05 uncorrected). Neither the present epileptiform EEG activity in the noADMCI patients may count on sleep onset or epileptiform EEG activity as “biological artifacts” in the EEG signal for the rsEEG source estimation (Traveling EEG slow waves from frontal cortex may have summed to local temporal delta rhythms during sleep). To avoid these confounding factors, we estimated EEG sources from rsEEG epochs taken during the first 3 min of the rsEEG recordings, paying attention that the noADMCI patients were in quiet wakefulness based on their posture in the armchair, general muscular tone, and on-going rsEEG traces during the experiments.

Furthermore, the selected EEG epochs for the source estimation were free from signs of sleep such as K complexes, sleep spindles, vertex shape waves, and slow waves during preliminary offline data analysis. Finally, rsEEG epochs were scrutinized to control for residual epileptiform EEG activity by two expert epileptologists. Keeping in mind the lack of effects of those possible causes of epileptiform EEG activity and abnormal rsEEG delta rhythms, a primary intrinsic neurophysiological cause of those activity may be hypothesized.

The present association between abnormalities in rsEEG source activities and epileptiform EEG activity in the noADMCI patients were more spatially and frequency restricted (i.e., temporal delta) as those abnormalities found in ADMCI patients with epileptiform EEG activity (i.e., central delta, parietal delta, occipital delta, temporal delta, and temporal theta) in unpublished data of our research group using the same EEG methodology. Taken together, these results suggest that epileptiform EEG activity in the present noADMCI patients may be related to primary neurophysiological abnormal mechanisms of hypersynchronization of cortical temporal neurons at delta frequencies. This background mechanism in the temporal lobe may be common to the ADMCI status. Therefore, it can be speculated that it might be provoked by several neuropathological processes occurring in pathological aging belonging to the MCI condition.

At this early stage of the research, we can just speculate about the primary pathophysiological mechanism at the basis of the abnormal temporal delta source activities in the noADMCI-EEA patients. This mechanism may produce exaggerated synchronization signals at delta frequencies in temporal cortico-thalamic and thalamocortical functional connectivity in quiet vigilance (42–47), maybe interfering with the vigilance regulation. In this speculative line, previous studies showed that a sleep deprivation partially deranged resting state cortical alpha and delta rhythms in healthy adults (48–51). These effects involved the temporal lobe and were partially recovered by taking a single dose of a pharmacological agent enhancing subjects' vigilance immediately after the sleep deprivation (48–51). Furthermore, those abnormal rsEEG rhythms due to the sleep deprivation in healthy adults were reminiscent of those observed in ADMCI, PDMCI, and LBDMCI patients resting in quiet wakefulness and not undergone to any sleep deprivation (27–29). Keeping in mind those data, the temporal lobe may represent a potential trigger of cortical neural hypersynchronization at delta frequencies and vigilance disturbances reacting to several kinds of neuropathological processes.

The above speculation is also in agreement with previous preclinical evidence collected in rodents. On one hand, rodent models of absence seizures (i.e., non-AD neuropathology) showed abnormal neural network properties, as revealed by quantitative EEG measurements, and seizures with generalized spike and wave discharges and cortical infarctions (52).

Furthermore, small cortical infarctions due to several neurobiological causes (i.e., non-AD neuropathology) resulted in recurrent epileptiform EEG activity and absence seizures in aged rats (52–54). On the other hand, transgenic mouse models with AD neuropathology pointed to abnormalities in ongoing EEG activity and epileptiform activity. Transgenic mice with APP and PS1 mutations inducing progressive accumulation of Abeta42 and diffuse plaques in the cerebral cortex, basal ganglia, and thalamus did manifest not only abnormalities in memory processes and ongoing cortical delta activity in the quiet wakefulness (55–58), but also seizures or subclinical, non-convulsive, and epileptiform EEG activity including cortical and hippocampal spikes and sharp waves (59–62).

### Methodological Remarks

In the interpretation of the current findings, the following methodological limitations should be considered.

The relatively small number of the noADMCI patients without (*N* = 19) and with (*N* = 13) epileptiform EEG activity did not permit a fine stratification of those patients based on demography and fine clinical features (e.g., history, clinical manifestations, etc.).

The clinical 10–20 electrode montage (i.e., 19 scalp electrodes) adopted for the rsEEG recordings is sub-optimal for the spatial sampling of rsEEG activity and the source estimation (63, 64). An optimal spatial sampling would have requested >64 scalp electrodes. Therefore, the current results should be considered as preliminary in the context of an exploratory and retrospective rsEEG study. However, in this exploratory context, the present findings may be acceptable for at least the two following reasons. First, rsEEG rhythms show largely dominant low spatial frequency components allowing valid anti-aliasing spatial sampling of instantaneous potential distributions by the mentioned 10-20 montage system using 19 scalp electrodes. This statement is grounded on many previous investigations carried out to study brain “microstates” in humans during the quiet wakefulness, defined as quasi-stable (i.e., few seconds) topographical patterns of rsEEG voltage maps (65). Indeed, these rsEEG topographic patterns are typically characterized by two very large voltage centroids (i.e., diameter of about ten centimeters), one of positive potentials and the other of negative potentials, changing position on the scalp over time (66, 67). These rsEEG topographic patterns, which are generated by widely distributed cortical neural networks revealed by resting-state functional MRI, can be recorded without spatial aliasing by the traditional 10-20 montage system (66, 67). Second, the present approach produces EEG findings that can be compared and discussed with those of several studies by independent research groups. They have successfully described abnormalities in rsEEG cortical sources estimated in patients with several cognitive and psychiatric disorders in whom rsEEG activity was recorded using the present 10-20 montage system and source estimation techniques [e.g., (68–74)]. In this line of reasoning, a Workgroup of International Federation of Clinical Neurophysiology expressed the consensus position that EEG source estimation of rsEEG data collected by the traditional 10-20 montage system can be used for exploratory, retrospective studies performed on available rsEEG databases relative to neurological and psychiatric patients (75). Those exploratory studies may provide informative preliminary results motivating further investments to develop cross-validating prospective rsEEG studies using EEG techniques with higher resolution (i.e., recordings based on 48–256 scalp electrodes, etc.).

Some rsEEG datasets were recorded using a relatively low sampling frequency of 128 Hz. We accepted those datasets to maximize the inclusion of a relatively large number of individual EEG datasets in Nold and noADMCI subjects. The rationale was our focus on delta (<4 Hz) and theta (4–8 Hz) source activities. However, the use of a 128-Hz sampling rate for the present EEG data analysis prevented the analysis of rsEEG sources at frequencies >40 Hz due to the aliasing effect. Future studies may use EEG datasets recorded with 256 Hz or higher frequency sampling to test additional hypotheses for gamma bands >40 Hz. In this regard, it should be noted that (Figure 4) unveiled that the magnitude of source activities at the beta and gamma (i.e., 14–40 Hz) bands was negligible in the present experimental conditions (i.e., eyes closed resting state and use of standard fixed frequency bands for beta and gamma). Therefore, it is not probable that relevant effects between noADMCI-noEEA and noADMCI-EEA may be observed at frequencies >40 Hz in the present experimental conditions.

We used the popular eLORETA freeware (http://www.uzh.ch/keyinst/loreta.htm) to estimate the cortical source activity generating scalp-recorded rsEEG rhythms (34). This freeware used a brain model based on a realistic cerebral shape taken from a template typically used in the neuroimaging studies, namely that of the Montreal Neurological Institute (MNI152 template). The use of an individual participant MRI rather than MNI could be of help in having a single model including rsEEG and atrophy, to see if epileptiform EEG activity might be an indirect epiphenomenon. Future studies should address this interesting issue.

The present experimental design did not allow testing the hypothesis that the presence of high values of rsEEG delta rhythms may increase the chance to produce EEA in noADMCI patients. Furthermore, that design did not allow testing the hypothesis that the presence of EEA may predict a worse disease progression and the disclosure of a clinical Epilepsy over time. In this respect, the present results motivate future long-term longitudinal studies in both noADMCI-EEA and noADMCI-noEEA participants to evaluate the kind of relationships between the magnitude of rsEEG delta rhythms, the manifestation and frequency of EEA, and clinical status during the disease progression. In those studies, proper regression and mixed linear models may test the significance and direction of those relationships between the magnitude of rsEEG delta rhythms and EEA. A fruitful example of a similar methodological approach is the PrESIDe study (76). In the PrESIDe study, a significant number of ADD patients (13–28%) were suspected of suffering from a sort of silent epilepsy (76). In the baseline recordings, the ADD patients with and without the suspect of epilepsy showed quite similar cognitive performances (76). However, those performances worsened more in the ADD patients with than without the suspicion of epilepsy at 1-year follow-up (76).

## Conclusions

In the present explorative and retrospective study, we investigated cortical sources of rsEEG rhythms in noADMCI patients with epileptiform EEG activity. The hypothesis was that those rsEEG sources might be more abnormal in noADMCI patients with epileptiform EEG activity as compared to noADMCI patients without that activity.

Results showed that 41% of the present noADMCI patients (*N* = 32) were characterized by that activity. Furthermore, the noADMCI-EEA patients showed higher temporal delta source activities as compared to the noADMCI-noEEA and Nold subjects. Those activities discriminated individuals of the two noADMCI groups with a moderate accuracy of about 70%. Notably, these results on rsEEG source activity depended neither on sleep signs and residual epileptiform EEG activity nor the clinical features, anthropometric features, cardiocirculatory markers, amyloidosis, tauopathy, amyloidosis, tauopathy, APOE genotyping, neurodegeneration, cerebrovascular lesions, and global cognitive status. Therefore, we speculated that in the prodromal stage of MCI not due to AD, epileptiform EEG activity may be found in a consistent percentage of cases (41%) and may be reflected by a primary derangement of neurophysiological temporal delta oscillatory mechanisms with possible effects on the regulation of quiet vigilance.

## Data Availability Statement

The datasets generated for this study are available on request to the corresponding author.

## Ethics Statement

All participants of the study in the subject gave their written informed consent under the World Medical Association's Declaration of Helsinki (as we reported in the paragraph Participants of the Materials and methods). This procedure was approved by the Ethics Committee of the following Clinical Units.

- Sapienza University of Rome, Rome, Italy.
- IRCCS SDN, Naples, Italy.
- Oasi Research Institute - IRCCS, Troina, Italy.
- IRCCS Hospital Policlinico San Martino, Genoa, Italy.
- University of Genoa, Genoa, Italy.
- IRCCS Istituto Centro San Giovanni di Dio Fatebenefratelli, Brescia, Italy.

## Author Contributions

CB, CDP, and EP contributed conception and design of the study. FT and MP organized the database. CDB and PC detected artifact-free EEG data. GN and RL performed the statistical analysis. GN and CDP wrote the first draft of the manuscript. All authors contributed to manuscript revision, read, and approved the submitted version.

## Conflict of Interest

The authors declare that the research was conducted in the absence of any commercial or financial relationships that could be construed as a potential conflict of interest.
